# Characterization of microRNA expression profiles in patients with intervertebral disc degeneration

**DOI:** 10.3892/ijmm.2013.1543

**Published:** 2013-10-30

**Authors:** BO ZHAO, QIANG YU, HAOPENG LI, XIONG GUO, XIJING HE

**Affiliations:** 1 Department of Orthopedics, The Second Affiliated Hospital, Xi’an Jiaotong University, Xi’an, Shaanxi 710004, P.R. China; 2 Key Laboratory of Environment and Gene Related Diseases, Department of Public Health, Xi’an Jiaotong University, Xi’an, Shaanxi 710061, P.R. China

**Keywords:** microRNA, intervertebral disc degeneration, spinal cord injury

## Abstract

Intervertebral disc degeneration (IDD) is associated with lower back pain and is a global burden with severe healthcare and socioeconomic consequences. However, the underlying mechanisms of IDD remain largely unelucidated. Accumulating evidence has indicasted that newly defined gene regulators, microRNAs (miRNAs), play a vital role in neurodegenerative, pathophysiological and certain reproductive disorders. To characterize the differential miRNA expression profiles between IDD and spinal cord injury, specimens from 3 patients with IDD and 3 with spinal cord injury were selected for microarray analysis. Total RNA from these 6 specimens was extracted and subjected to global miRNA expression analysis using the Exiqon miRCURY™ LNA Array (v.16.0). The microarray data were then validated by quantitative reverse transcription polymerase chain reaction (qRT-PCR). In addition, bioinformatics analysis was performed to investigate the dysregulated miRNA target genes and signaling pathways involved. Among the miRNAs analyzed, 25 miRNAs were found to be upregulated and 26 were found to be downregulated in the IDD group compared with the spinal cord injury group. The qRT-PCR results validated the microarray data. Bioinformatics analysis indicated that the signaling pathways most likely to be controlled by these miRNAs were the phosphoinositide 3-kinase (PI3K)-Akt, mitogen-activated protein kinase (MAPK), epidermal growth factor receptor (EGFR; ErbB) and Wnt pathways. Our results demonstrated that the miRNA expression in patients with IDD differed significantly from that in patients who sustained injury to the intervertebral disc. Our data indicate that the dysregulated miRNAs control the signaling pathways important for the maintenance of IDD. Further studies on miRNA target gene identification and biological functions may address the specific regulatory mechanisms of miRNAs in IDD, and may provide valuable insight into the diagnosis and treatment of IDD.

## Introduction

Intervertebral disc degeneration (IDD) is the main cause of lower back pain, and is a medical condition that places a heavy burden on the global healthcare system with severe socioeconomic consequences (1–3). Due to the nature of the risk factors for IDD, including family history, lumbar load and workload (4), the incidence of IDD is higher in developing countries, particularly in China. To date, it is agreed that IDD is similar to other complex diseases since its etiology has hereditary and environmental influences, both of which generate a small overall contribution to the relative risk. The main pathological changes that occur in IDD involve the excessive apoptosis of intervertebral disc cells and the components of the extracellular matrix (ECM). Although a number of studies have focused on the etiology of IDD, such as genetics (5), mechanical load (6), and environmental factors (7), the underlying pathology is not yet fully understood.

microRNAs (miRNAs) are a type of small non-coding RNA molecules of 20–22 nucleotides in length and were first reported in *C. elegans*(8,9). miRNAs function by the partial or completely binding to the 3′-untranslated region (UTR) of their target mRNAs, and thereby trigger either translation inhibition or mRNA degradation (10,11). A single miRNA is capable of regulating the expression of several target genes, whereas a single target gene can also be modulated by several miRNAs (12). On a molecular level, miRNAs have been shown to act as key regulators in a wide variety of biological processes, such as cell growth, differentiation, resistance to chemotherapeutic drugs and organ development (13,14). Furthermore, miRNAs play a central role in cancer, as well as in inflammatory, neurodegenerative, pathophysiological and certain reproductive disorders (15,16). In a previous study, it was demonstrated that the aberrant expression of miRNA-140 was associated with the degenerative disease, osteoarthritis (OA), which is characterized by pathological changes similar to those which occur in IDD (17). miRNA-140 has also been shown to regulate cartilage development and homeostasis by targeting insulin-like growth factor binding protein-5 (IGFBP-5), Smad family member 3 (Smad3) and Adam metallopeptidase with thrombospondin type 1 motif, 5 (Adamts-5) (18–20). Several other miRNAs, such as miR-34a (21), miR-21 (22) and miR-675 (23) have also been found to be associated with chondrocyte apoptosis, proliferation or cartilage matrix production. Moreover, miR-155, a well-documented miRNA involved in various diseases (24), has been reported to promote Fas-mediated apoptosis by targeting Fas-associated protein with death domain (FADD) and caspase-3 in human IDD (25). These findings demonstrate the effectiveness of miRNAs as regulators for determining the pathogenesis of degenerative disorders, such as IDD.

The aim of the present study was to isolate miRNAs from patients with IDD and spinal cord injury and subsequently identify the differential miRNA expression profiles between them. Bioinformatics analysis was then performed to investigate the dysregulated miRNA target genes and the signaling pathways involved, which may enhance our understanding of the molecular mechanisms leading to IDD.

## Materials and methods

### Sample collection

The study was approved by the Human Ethics Committees Review Board at Xi’an Jiaotong University, Xi’an, China and written informed consent was obtained from each patient prior to enrollment.

Patients presenting with lumbar intervertebral disc herniation (LIDH), a medical condition that is representative of IDD, were selected as the experimental subjects [experimental group (EG)]. The EG consisted of 20 individuals with severe clinical symptoms. Disc degeneration was confirmed by both magnetic resonance imaging (MRI) with an apparent decrease in the T2-weighted signal, as well as hematoxylin and eosin (H&E) staining with obvious morphological changes. Disc specimens were classified as grade IV (IDD group) or grade I (spinal cord injury group) according to the MRI results (26). Patients with degenerative spinal stenosis, idiopathic scoliosis, tumors, infections, or previous lumbar disc surgery were excluded from this study.

The control group (CG) included 20 individuals who received surgical treatment within 6 h after sustaining injury. Their medical histories showed no evidence of pre-existing spinal disorders, disc degeneration or previous spine-related surgeries. This information was confirmed by both MRI and H&E staining of the tissue samples. The nucleus pulposus (NP) tissues were carefully dissected during surgery and subsequently subjected to various methods of analysis, according to the corresponding procedures. Briefly, the whole tissues were rinsed with phosphate-buffered saline (PBS, pH 7.2) and then separated into 2 sections. One half was snap-frozen and stored in liquid nitrogen within 30 min after removal from the patient, with subsequent storage at −80ºC, while the remaining half was fixed with paraformaldehyde.

### H&E and TUNEL staining

Standardized H&E staining was used to evaluate the morphology of NP tissue in the paraffin-embedded samples. To identify apoptosis in NP cells, TUNEL staining was performed using the TUNEL apoptosis assay kit according to the manufacturer’s instructions (Roche, Basel, Switzerland). The results were obtained using an optical microscope.

### RNA extraction and quality inspection

Total RNA from each sample was individually isolated using TRIzol reagent (Invitrogen, Carlsbad, CA, USA) and the miRNeasy mini kit (Qiagen, Valencia, CA, USA) according to the manufacturer’s instructions. This procedure efficiently recovered all RNA species, including miRNAs. RNA quality and quantity were measured using a NanoDrop spectrophotometer (ND-1000; NanoDrop Technologies, Wilmington, DE, USA) and RNA integrity was determined by gel electrophoresis.

### RNA labeling and array hybridization

Following RNA isolation, the miRCURY™ Hy3™/Hy5™ Power labeling kit (Exiqon, Vedbaek, Denmark) was used for miRNA labeling according to the manufacturer’s instructions. Each 1 μg of sample was 3′-end-labeled with Hy3 fluorescent label using T4 RNA ligase. After the labeling procedure was terminated, the Hy3-labeled samples were hybridized to the miRCURY LNA Array (v.16.0) (Exiqon) according to the manual provided with the array. The total mixture with hybridization buffer was hybridized to the microarray in a 12-Bay Hybridization System (Hybridization System; NimbleGen Systems, Inc., Madison, WI, USA), which provides an active mixing action and a constant incubation temperature to improve hybridization uniformity and enhance the signal. Following hybridization, the slides were washed several times using the wash buffer kit (Exiqon), and finally dried by centrifugation. The slides were then scanned with the Axon GenePix 4000B microarray scanner (Axon Instruments, Foster City, CA, USA), which contains >1,891 capture probes annotated in miRBase 16.0 and 66 additional new miRPlus™ human microRNAs that are proprietary and not found in miRBase. Our microarray data were MIAME compliant and have been deposited in the MIAME compliant database GEO (Accession no. GSE45856).

### Array data analysis

Scanned images were imported into GenePix Pro 6.0 software (Axon) for grid alignment and data extraction. Replicate miRNAs were averaged, and miRNAs with intensities ≥50 in all samples were selected to calculate the normalization factor. The expressed data were normalized using the median normalization method. Following normalization, significantly differentially expressed miRNAs were identified through volcano plot filtering. Hierarchical clustering was performed using MEV software (v4.6, TIGR). The miRNA was defined as being differentially expressed between the compared groups if the P-value was <0.05 and the fold change above 2.

### Quantitative reverse transcription polymerase chain reaction (qRT-PCR)

Seven miRNAs from the array data analysis were selected for validation using the SYBR-based qPCR method. The 7 target miRNAs selected belonged to one of the two following categories: i) miRNAs associated with chondrocyte apoptosis and ECM degeneration (miR-34a and miR-675^*^); and ii) miRNAs with particularly high fold changes in expression according to the microarray results (miR-10a^*^, miR-25^*^, miR-182, miR-130b^*^ and miR-200c). Total RNA (100 ng) was reverse transcribed to cDNA using miRNA-specific stem-loop RT primers in a GeneAmp PCR System 9700 (Applied Biosystems, Foster City, CA, USA). Quantitative PCR was performed using SYBR-Green (Invitrogen) according to the manufacturer’s instructions in a Rotor-Gene 3000 Real-time PCR instrument (Corbett Research, Brisbane, Australia). The miRNA levels were normalized to U6 as an internal control. The relative abundance of each miRNA was calculated using the comparative Ct (2^−ΔΔCt^) method, and the results were assessed by a t-test.

### Bioinformatics analysis

The 3 most popular databases, TargetScan (27), miRanda (28) and miRDB (29), were used to predict the target genes of the differentially expressed miRNAs. To reduce the false positive results, the genes predicted by at least 2 of these 3 databases were selected as differential miRNA targets for further analysis. Specifically, the target genes for the star form of miRNAs (miRNA^*^) were predicted by both the miRanda and miRDB databases, whereas the target genes for the non-star form of miRNAs (miRNA) were predicted by the miRanda and TargetScan databases. For functional annotation analysis, the DAVID database (30) was used to annotate the function of target genes in the module. The GO terms with adjusted P-value <0.05 and count <2 were selected. The target genes were further put into the KEGG database (31) to identify the enriched pathways. The count number <2 and P-value >0.05 were selected as the cut-off criteria.

### Statistical analysis

Comparisons of 2-group parameters were performed using the Student’s t-test. Comparisons of multiple group data were performed using one-way analysis of variance followed by Turkey’s post hoc test. A value of P<0.05 was considered to indicate a statistically significant difference. Statistical analysis was performed using the SPSS statistical software package (SPSS Inc., Chicago, IL, USA).

## Results

### Basic patient information

Patients with LIDH were selected as the EG. This group consisted of 10 males and 10 females, ranging from 38 to 68 years in age with an average age of 53.9±8.5 years. In this group, the degenerative intervertebral disc segments were the L4/5 segment (12 patients), L5/S1 segment (7 patients), and L3/4 segment (1 patient). According to the MRI results, 14 specimens were classified as grade V and 6 as grade IV. By contrast, patients with spinal cord injury were selected as the CG, which included 11 males and 9 females, ranging from 24 to 55 years in age with an average age of 41.1±9.0 years. In this group, the degenerative intervertebral disc segments were the L4/5 segment (8 patients), L3/4 segment (3 patients), L2/3 segment (3 patients) and the L1/2 segment (6 patients), and 16 were classified as grade II and 4 as grade I based on an MRI. Three patients from each of these 2 groups were randomly selected for microarray analysis. The basic information of these patients is presented in Table I.

### Histomorphological analysis of LIDH and spinal cord injury specimens

The intervertebral disc has a unique structure, with a gelatinous, amorphic NP surrounded by a highly organized annulus fibrosus. The ECM, which is produced and maintained by chondrocytic NP cells, is primarily composed of proteoglycans within a type II collagen scaffold (32).

**Figure 1:**
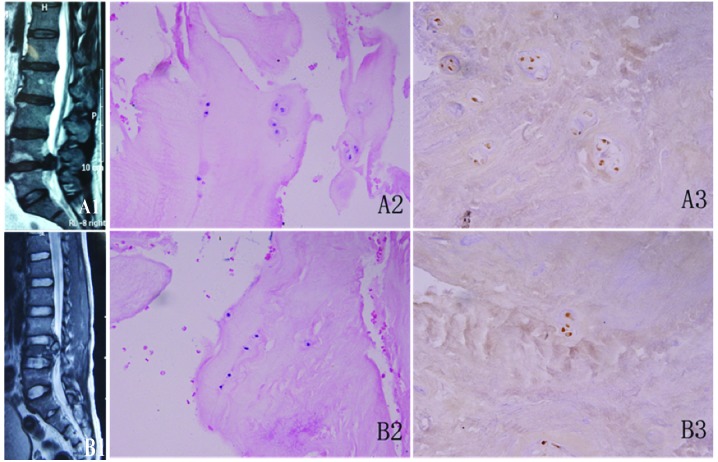
Characteristics of lumbar intervertebral disc herniation and spinal cord injury. (A1) L4/5 disc herniation. The intervertebral disc was classified as grade IV according to magnetic resonance imaging (MRI). (B1) L4 vertebral burst fracture. The intervertebral disc was classified as grade I according to MRI. Hematoxylin and eosin staining of nucleus pulposus tissue from a patient with intervertebral disc degeneration (A2) and a patient with spinal cord injury (B2); TUNEL staining of nucleus pulposus from a patient with intervertebral disc degeneration (A3) and a patient with spinal cord injury (B3) (original magnification, ×400).

To confirm the intervertebral disc degeneration and spinal cord injury, the specimens were subjected to H&E staining. The NP tissue of the lumbar intervertebral disc showed the following features under light microscopy: the NP cells which appeared as round, chondrocyte-like cells were the only cellular structures observed, the cytoplasm was stained red, the nucleus was stained blue-black and the ECM was stained light red (Fig. 1). In the spinal cord injury group, there was a higher number of NP cells, most of which were isolated in the cartilage lacunae; individual NP cells appeared in pairs or small cell clusters, and only a few empty lacuna (no chondrocyte-like cells were present) were observed (Fig. 1B2). In the IDD group, the NP cells whose nuclei had become small were sparse, more cell clusters or multinucleated giant cells were present and the frequency of empty lacunae increased (Fig. 1A2).

TUNEL staining was then performed on the NP tissue samples from the EG. Fewer chondrocyte-like cells were observed, and there were many TUNEL-positive cells with brown-stained nuclei (Fig. 1). These cells also exhibited changes in nuclear morphology, fragmentation or chromatin margination (Fig. 1A3). In the CG, on the other hand, more chondrocyte-like cells and fewer TUNEL-positive cells were present (Fig. 1B3).

### miRNAs are differentially expressed in IDD

**Figure 2:**
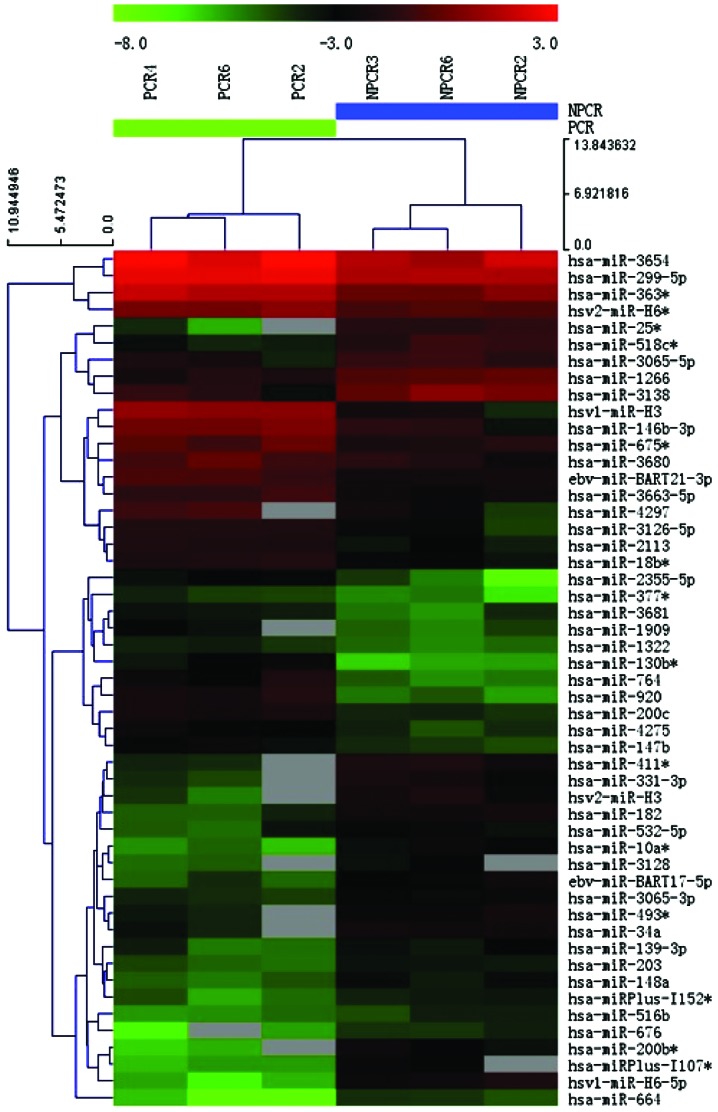
Heat map and hierarchical clustering. Heat map shows the results of two-way hierarchical clustering of miRNAs and samples. PCR2, PCR4 and PCR6 represent the lumbar intervertebral disc herniation (LIDH) patient samples, whereas NPCR2, NPCR3 and NPCR6 represent the spinal cord injury samples. Each row represents a miRNA and each column represents a sample. The miRNA clustering tree is shown on the left, and the sample clustering tree appears at the top. The color scale shown at the top illustrates the relative expression level of a miRNA in the certain slide: red, high relative expression level; green, low relative expression level.

The 6th generation of miRCURY LNA Array (v.16.0) (Exiqon) employed in this study contained >1,891 capture probes, covering all human, mouse and rat miRNAs annotated in miRBase 16.0, as well as all viral miRNAs related to these species. This array also consisted capture probes for 66 new miRPlus human miRNAs that are proprietary and not found in miRBase 16.0. After performing fold change filtering (fold change ≥2) on the differentially expressed miRNAs, we found that 54 miRNAs were upregulated and 53 miRNAs were downregulated in the IDD group compared with spinal cord injury group. Volcano plot filtering was then performed to identify the significantly differentially expressed miRNAs between these 2 groups. The thresholds for screening differentially expressed miRNAs were a fold change of ≥2 and a P-value of ≤0.05. The heat map (Fig. 2) indicated the results of a two-way hierarchical clustering of miRNAs and samples. A total of 51 miRNAs exhibited a significant difference in expression in the IDD group. Amongst these miRNAs, 25 miRNAs showed an upregulated expression (Table II), whereas 26 miRNAs showed a downregulated expression (Table III) in the IDD group compared with the spinal cord injury group.

**Figure 3:**
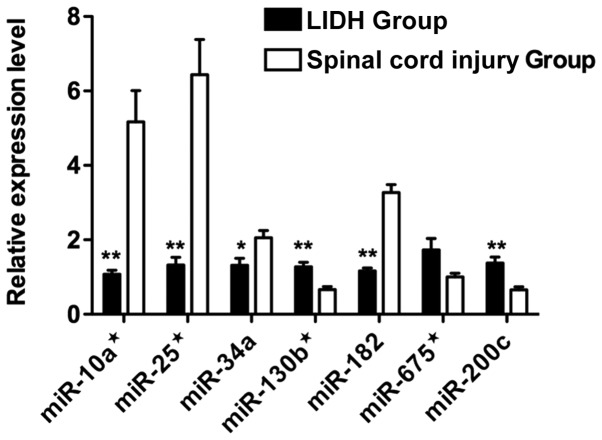
Confirmation of microarray results by qRT-PCR in samples from the lumbar intervertebral disc herniation (LIDH, experimental) and spinal cord injury (control) groups. All the microRNAs apart from miR-675^*^ showed statistically significantly changes in expression in the experimental group compared with the control group. ^*^P<0.05 and ^**^P<0.01.

Based on their expression levels and fold difference in expression, 3 upregulated miRNAs (miR-130b^*^, miR-675^*^ and miR-200c) and 4 downregulated miRNAs (miR-10a^*^, miR-25^*^, miR-34a and miR-182) were selected for validation by qRT-PCR. All of the miRNAs examined, with the exception of miR-675^*^, showed a statistically significant difference in expression in a manner consistent with the data from microarray analysis (P<0.05) (Fig. 3).

### Bioinformatics analysis

**Figure 4:**
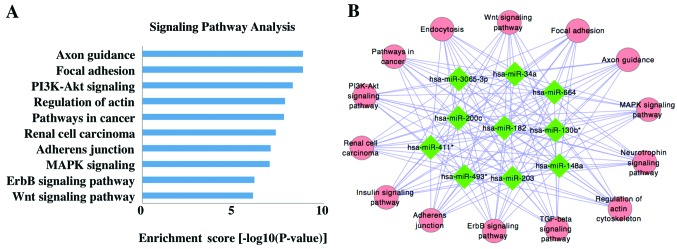
(A) Top 10 signaling pathways predicted to be regulated by the differentially expressed miRNAs. (B) Network map of miRNAs and signaling pathways involved.

**Figure 5:**
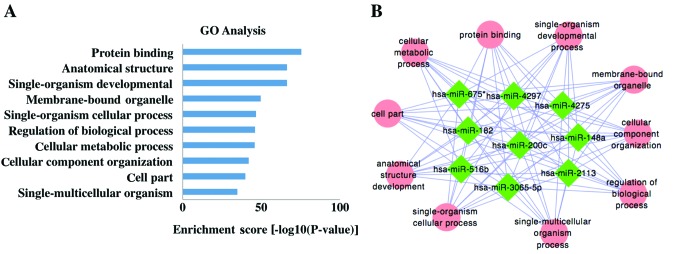
(A) Top 10 functional GO terms of the predicted miRNA target genes. (B) Network map of miRNAs and GO terms involved.

Since miRNAs function by targeting mRNAs, we retrieved the putative target genes of differentially expressed miRNAs from 3 databases and selected the target genes retrieved by at least 2 databases. The target genes were then subjected pathway enrichment analysis using KEGG pathways to find the canonical pathways controlled by the identified miRNAs. Among the top 10 signaling pathways mostly likely to be regulated by the miRNAs were the phosphoinositide 3-kinase (PI3K)-Akt, mitogen-activated protein kinase (MAPK), epidermal growth factor receptor (EGFR; ErbB) and Wnt pathways (Fig. 4A). The network between the miRNAs and signaling pathways is illustrated in Fig. 4B. The function of the target genes was then predicted by the GO enrichment analysis. The predicted target genes were principally enriched for GO terms related to processes, such as protein binding and anatomical structure (Fig. 5A). The network miRNAs involved were presented in Fig. 5B.

## Discussion

In the present study, 3 samples were selected from each of the LIDH and spinal cord injury groups. Their miRNA expression patterns were quantified and we then evaluated the differences in their respective miRNA expression profiles by microarray analysis. We demonstrated that 25 miRNAs were upregulated and 26 miRNAs were downregulated in the IDD group compared with the spinal cord injury group. Given the potential for false positives with microarray technology, as well as its inherent limitations as regards sensitivity and quantification, qRT-PCR was then performed to validate the microarray data. However, microarray expression analysis is still a powerful, high-throughput and versatile tool for the study of genome-wide miRNA expression profiles. Therefore, microarray technology was used in this study to preliminarily screen the differentially expressed miRNAs, potentially shedding light on the regulatory mechanisms of miRNAs in IDD.

Since NP tissue cannot be separated from completely normal living bodies to serve as normal controls, the controls are usually selected from the following 2 groups when investigating differential miRNA expression profiles in patients with IDD: i) cases in which the NP tissue is detached from the body immediately following accidental death; ii) patients with spinal disease requiring discectomy, such as those with congenital scoliosis or severe spinal injury. For the former group, NP tissue may be obtained through osteotomy, whereas in the latter group, NP tissue can be acquired via discectomy and fusion fixation. In all selected cases, it is imperative to exclude other spinal diseases and other underlying diseases that may cause changes in gene expression. In this study, we selected patients with spina cord injury as the CG for the following reasons: i) separating NP tissue from accidental death victims is very difficult as it is not easy to verify whether the intervertebral disc has degraded, and there are various changes in gene expression following accidental death; ii) congenital scoliosis is a congenital disorder, and given that the local lesion was stimulated by abnormal stress for a long period of time, it is likely that its gene expression differs from that in normal healthy individuals; iii) there were a number of severe spinal injuries needing discectomy and fusion fixation in our clinic, and thus it was convenient to obtain the NP tissue from these patients. Changes due to local inflammation and associated post-trauma reactions in the intervertebral disc should be taken into consideration when analyzing the results. It is noted that we cannot entirely exclude the possibility that the LIDH and spinal cord injury groups had different genetic backgrounds.

Susceptibility to IDD is greater in individuals with particular alleles, such as the Trp2 allele of COL9A2 (33) or different genetic polymorphisms in vitamin D receptor (VDR) (34). Following gene sequence alignment and target gene prediction using the 3 popular databases (PicTar, TargetScan and miRanda), the COL9A2 gene was found to be a putative target gene of miR-146b-3p, which was upregulated by 3.80-fold in the degenerative disc (P=0.003). Moreover, miR-146 has been reported to target several other mRNAs, including apoptosis-related genes, such as FADD (35), inflammation-associated genes, such as interleukin (IL)-1β, IL-6 and tumor necrosis factor (TNF) (36), as well as metabolism-related genes, such as matrix metalloproteinase (MMP)-16 (37). Given that the abovementioned biological processes and genes are involved in the pathological changes observed in IDD, we predicted that miR-146 may play an important role in the occurrence and development of IDD.

A previous study demonstrated that silencing miR-34a can effectively reduce IL-1β-induced apoptosis in rat chondrocytes (21). In this study, miR-34a was found to be downregulated by 0.45-fold (P<0.05) in degenerative NP compared with the spinal cord injury group. This result was further confirmed by qRT-PCR (0.64-fold, P<0.05). One probable explanation is that spinal cord injury may induce the apoptosis of intervertebral disc cells through a caspase-dependent pathway. Thoracolumbar fractures can induce early caspase-dependent apoptosis in disc cells of the affected intervertebral disc, in part by downregulating the anti-apoptotic protein, Bcl-2, as well as signaling via the death receptor complex [TNF receptor (TNFR) I and Fas receptor (FasR)] (38). As previously demonstrated, compared with degenerative intervertebral discs, traumatic thoracolumbar intervertebral discs have an increased number of TUNEL-positive cells, which is evidence of apoptosis involving both receptor-mediated and mitochondrial-dependent pathways (39). In this study, the spinal cord injury group samples were obtained upon surgery performed on patients with spinal cord injury that took place within 6 h following injury. The morphological changes in our spinal cord injury group samples were the same as those observed in the normal NP tissue. Based on these data we hypothesized the following: soon after spinal injury, the expression of miR-34a is upregulated to a level even higher than that observed during degeneration, resulting in a large number of cells undergoing apoptosis and further promoting intervertebral disc degeneration.

In the present study, the differentially expressed miRNAs were predicted to control several pathways relevant for the regulation of IDD. It has been demonstrated that the transcriptional activation of the PI3K-Akt pathway is involved in lumbar disc degeneration (40). As shown in a previous study, hyperbaric oxygen treatment suppresses the MAPK signaling pathway in degenerated human intervertebral disc cells (41). In addition, investigators from The Netherlands have used a canine model of IDD to examine the biochemical changes associated with chondroid metaplasia, and found a downreguation of Wnt signaling and caveolin-1 expression (42). Our results suggest that miRNAs are important regulators of IDD through the modulation of several signaling pathways.

In conclusion, our results demonstrated that 25 miRNAs were upregulated and 26 were downregulated in the NP tissue of LIDH patients compared with the patients spinal cord injury. Bioinformatics analysis predicted the target genes and signaling pathways of these miRNAs, which may enhance our understanding of the involvement of miRNAs in the occurrence and development of IDD. Further studies on miRNA functions and target gene verification would provide an experimental basis for the diagnosis and treatment of IDD, which remains an important area for future investigation.

## Figures and Tables

**Table I:** Basic patient information.

Sample ID (EG/CG)	LIDH patients (EG)				Spinal cord injury patients (CG)			
	
	Age	Gender	Segment	MRI grade	Age	Gender	Segment	MRI grade
PCR2/NPCR2	63	M	L4/5	V	56	F	L4/5	II
PCR4/NPCR4	70	M	L4/5	V	51	M	L3/4	II
PCR6/NPCR6	55	F	L4/5	IV	58	M	L4/5	II

Lumbar intervertebral disc herniation (LIDH) patients in the experimental group (EG) and spinal cord injury patients in the control group (CG) were used for microRNA microarray analysis. Basic patient information including age, gender, spinal segment and magnetic resonance imaging (MRI) grade was collected. PCR2, PCR4 and PCR6 represent the lumbar intervertebral disc herniation (LIDH) patient samples, whereas NPCR2, NPCR3 and NPCR6 represent the spinal cord injury samples F, female; M, male.

**Table II:** Upregulated microRNAs in intervertebral disc degeneration compared with spinal cord injury.

ID	Name	Fold change	P-value
147900	hsv2-miR-H6*	2.012	0.011
148599	has-miR-3680	2.335	0.047
17411	hsa-miR-147b	2.491	0.010
42932	hsa-miR-920	8.215	0.005
145990	ebv-miR-BART21-3p	2.702	0.003
146058	hsv1-miR-H3	9.582	0.000
42787	hsa-miR-130b*	6.266	0.005
147743	hsa-miR-4275	2.374	0.009
148633	hsa-miR-299-5p	2.155	0.000
42461	hsa-miR-146b-3p	3.798	0.003
146096	hsa-miR-764	6.153	0.037
148597	hsa-miR-3663-5p	2.497	0.006
145826	hsa-miR-18b*	2.391	0.002
42899	hsa-miR-377*	2.496	0.021
147800	hsa-miR-2355-5p	4.078	0.005
148353	hsa-miR-3681	2.228	0.031
42859	hsa-miR-675*	2.462	0.025
147925	hsa-miR-3126-5p	2.662	0.005
148379	hsa-miR-3654	2.258	0.041
17427	hsa-miR-200c	3.330	0.001
27544	hsa-miR-363*	2.668	0.005
146179	hsa-miR-2113	2.877	0.000
146180	hsa-miR-1909	2.861	0.007
147632	hsa-miR-4297	3.904	0.007
46408	hsa-miR-1322	2.186	0.010

**Table III:** Downregulated microRNAs in intervertebral disc degeneration compared with spinal cord injury.

ID	Name	Fold change	P-value
28019	hsa-miR-10a^*^	0.194	0.003
146077	hsv2-miR-H3	0.204	0.041
147654	hsa-miR-3138	0.260	0.017
145973	hsa-miR-664	0.176	0.003
46818	ebv-miR-BART17-5p	0.298	0.008
17624	hsa-miR-532-5p	0.431	0.045
145821	hsa-miR-518c^*^	0.206	0.005
10975	hsa-miR-182	0.248	0.001
148471	hsa-miRPlus-l152^*^	0.400	0.006
11004	hsa-miR-203	0.424	0.001
145974	hsa-miR-200b^*^	0.121	0.027
42451	hsa-miR-139-3p	0.448	0.047
42929	hsa-miR-25^*^	0.110	0.003
147682	hsa-miR-H6-5p	0.080	0.004
148393	hsa-miR-676	0.242	0.016
147536	hsa-miRPlus-l107^*^	0.150	0.004
10955	hsa-miR-148a	0.360	0.004
46517	hsa-miR-1266	0.370	0.004
147903	hsa-miR-3065-3p	0.456	0.004
11125	has-miR-493^*^	0.449	0.042
42784	hsa-miR-411^*^	0.275	0.028
42887	hsa-miR-331-3p	0.275	0.040
148033	hsa-miR-3065-5p	0.443	0.030
27217	hsa-miR-34a	0.455	0.005
11151	hsa-miR-516b	0.420	0.029
147545	hsa-miR-3128	0.307	0.029
